# Prevalence and Morphometric Features of the Canalis Sinuosus and Its Accessory Canals in Turkish Adult Patients: A Single-Center Cross-Sectional CBCT Study

**DOI:** 10.3390/diagnostics16213437

**Published:** 2026-10-22

**Authors:** İsmail Çapar, Emre Haylaz, Çiğdem Şeker, Gediz Geduk, Fahrettin Kalabalık, Suayip Burak Duman

**Affiliations:** 1 Department of Oral and Maxillofacial Radiology, Faculty of Dentistry, Zonguldak Bülent Ecevit University, Zonguldak 67600, Türkiyegedizgeduk@gmail.com (G.G.); 2 Department of Oral and Maxillofacial Radiology, Faculty of Dentistry, Sakarya University, Sakarya 54100, Türkiye; 3 Department of Oral and Maxillofacial Radiology, Faculty of Dentistry, İnönü University, Malatya 44000, Türkiye; 4 Department of Diagnostic Sciences, Texas A&M College of Dentistry, Dallas, TX 75226, USA

**Keywords:** canalis sinuosus, accessory canal, cone-beam computed tomography, anterior maxilla, anatomical variation, morphometry

## Abstract

**Background/Objectives**: The canalis sinuosus (CS) and its accessory canals (ACs) are neurovascular canals of the anterior maxilla at risk during surgery. We examined their prevalence, side distribution, diameter, localization, and course on cone-beam computed tomography (CBCT) and explored associations with age and sex. **Methods**: Two observers independently read CBCT images of 603 adults from a single center. Canals were recorded only if they measured at least 0.5 mm, and ACs only if their continuity with the CS could be traced. Age, sex, and side were examined with generalized estimating equations and Benjamini–Hochberg correction. **Results**: The CS was present in 53.2% of patients, bilaterally in 97.5% of them. ACs were seen in 59.5% of CS-positive patients (31.7% overall). Most ended in the lateral incisor region (26.5%) and ran straight vertically (63.4%). In exploratory analyses, age was associated with the presence of both canals (*q* = 0.040), whereas sex and side showed no significant association; the CS was wider in men (*β* = 0.227 mm; *q* < 0.001). **Conclusions**: At least one AC was present in 31.7% of patients, and 43.4% of ACs ended at lateral incisor or canine sites. When CBCT is available before anterior maxillary surgery, the planned site should be examined for these canals; demographic associations need confirmation.

## 1. Introduction

The canalis sinuosus (CS) is an intraosseous canal that carries the anterior superior alveolar nerve and its accompanying vessels through the anterior maxilla. Wood Jones described it in 1939: it leaves the infraorbital canal and follows a winding path along the lateral wall of the nasal cavity before reaching the anterior maxillary region [1,2]. Its terminal branches, and the accessory canals (ACs) that arise from it, may run toward the alveolar process and the palatal cortical plate, most often in the incisor and canine regions, where they contribute to the neurovascular supply of the area [3,4].

For decades the nasopalatine canal was treated as the only neurovascular structure of any consequence in the anterior maxilla. That view has changed. The CS and its accessory canals are now known to be at risk during implant placement, bone grafting, orthognathic and endodontic surgery, and the removal of impacted teeth [5,6,7,8]. Damage to them has been linked to bleeding, pain, paresthesia, sensory disturbance, epistaxis, and prolonged postoperative discomfort [9,10,11]. On periapical and panoramic films an accessory canal may also be mistaken for a periapical lesion or another radiolucency, which occasionally leads to unnecessary treatment [5,8,12].

The canal is nevertheless easy to miss in routine practice. It is narrow, its course varies between individuals, and two-dimensional radiographs suffer from superimposition and limited spatial resolution [8,12,13]. Cone-beam computed tomography (CBCT) avoids both problems and has become the method of choice for studying the CS and its branches [10,13].

CBCT studies of the past decade report wide differences in how often these canals are seen and in their morphology, localization, diameter, and course [11,14]. Prevalence figures differ several-fold between series, and the imaging protocol, voxel size, slice thickness, diagnostic criteria, and composition of the sample all play a part [11,13]. Some authors have argued that the CS should be regarded as a normal component of the anterior maxilla rather than as a rare variation [14]. Accessory canals have likewise been described with varying orientations and end points, concentrated in the lateral incisor and canine regions, which are also the regions most often chosen for implants for esthetic and anatomical reasons [9].

Detailed morphometric description of these canals has repeatedly been recommended as a way of reducing iatrogenic injury [7,9,15]. Reported differences concern not only prevalence but bilateral distribution, orientation, and the region in which a canal ends [14,15,16]. A higher frequency in men, a relationship with alveolar bone volume, and a predominantly curved canal morphology have all been described [14,16,17], whereas the influence of age and sex on morphometric measurements remains unsettled [14,15].

Part of this inconsistency is methodological. Samples differ, as do imaging protocols, voxel size, the smallest diameter accepted, the criteria used to identify a canal, and the morphological classification applied. Many studies have also examined a limited set of features, usually of either the main canal or its accessory branches [4,7,8,10,12,13,15]; prevalence, side distribution, morphometry, course, terminal localization, and demographic associations have rarely been reported together for the same patients. Direct comparison between series is therefore difficult, and large samples assessed against stated criteria are still needed.

A prevalence figure means little on its own unless the criteria behind it are stated. We therefore set out to describe the CS and its accessory canals in a large clinical CBCT sample, using an explicit detectability threshold and an explicit requirement of anatomical continuity between an accessory canal and the main canal, and to relate prevalence, morphometry, side distribution, course, and terminal localization to age and sex. Because both sides of every patient were assessed, the analyses take within-patient dependence into account, and accessory canal frequency is given for the whole sample and for CS-positive patients separately. The study adds to earlier series in three respects: all of these features were recorded in the same patients under explicit criteria; accessory canal frequency is reported in a form that can be compared across series with different CS detection rates; and the pairing of sides and the number of comparisons are handled explicitly in the analysis. The associations with age and sex were examined as exploratory questions, and the surgical relevance of the findings is discussed as an inference from anatomy rather than as a tested outcome.

## 2. Materials and Methods

### 2.1. Study Population and Eligibility Criteria

In this retrospective cross-sectional study, we reviewed the CBCT records of patients examined at the Department of Oral and Maxillofacial Radiology, Faculty of Dentistry, Zonguldak Bülent Ecevit University (Zonguldak, Western Black Sea region of Türkiye) between 2021 and 2024. All 927 examinations held in the institutional database for that period were screened consecutively against the eligibility criteria. They had been requested for a variety of reasons: implant planning, impacted teeth, suspected pathology, surgical and endodontic assessment, and other diagnostic questions involving the maxillofacial region.

**Figure 1:**
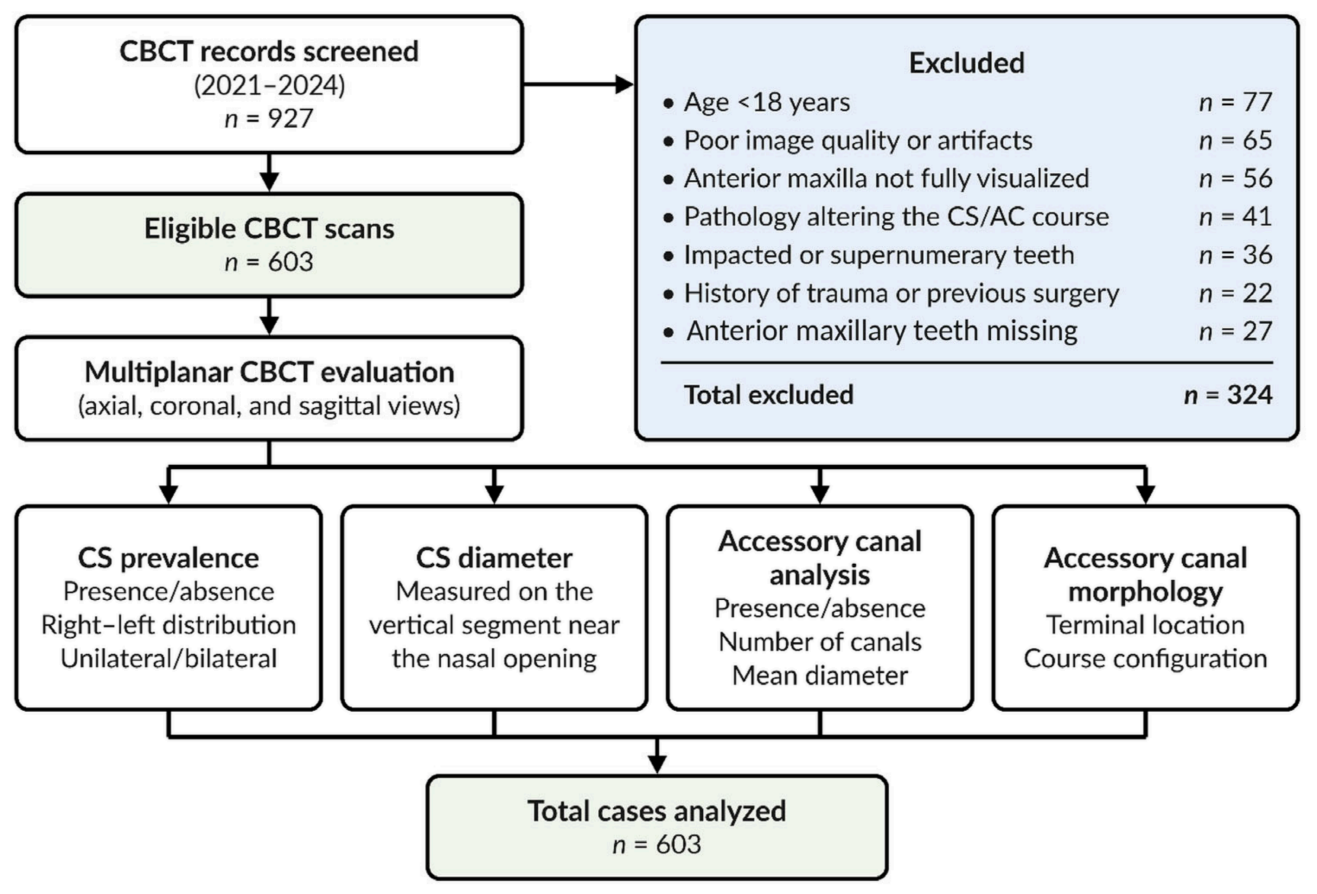
Workflow of retrospective CBCT selection and evaluation of canalis sinuosus and accessory canals. Exclusion reasons were applied in the order listed, and each excluded record was counted under the first reason that applied. All counts (*n*) refer to CBCT examinations, one per patient; “anterior maxillary teeth missing” denotes a missing incisor, canine, or first premolar. CS, canalis sinuosus; AC, accessory canal.

Of these, 324 records were excluded. Exclusion reasons were applied hierarchically, in the order in which they are listed here and in Figure 1; when more than one reason applied, only the first in this order was recorded, so each excluded record was counted once: age under 18 years (77), insufficient image quality or artifacts (65), incomplete imaging of the anterior maxilla (56), pathology capable of displacing the canalis sinuosus or its accessory canals (41), impacted or supernumerary teeth (36), previous trauma or surgery (22), and a missing incisor, canine, or first premolar, which made the tooth-referenced localization scheme inapplicable (27). Because only one reason was recorded per record, co-existing reasons cannot be reconstructed, and every count after the first is a lower bound for the frequency of that condition among the screened records. Patients younger than 18 years were excluded because the maxilla is still growing in childhood and adolescence, unerupted permanent teeth, the canines in particular, occupy the region traversed by the CS and its branches in younger patients, and implant placement, the procedure for which these canals matter most, is generally deferred until growth is complete [18]. Accessory canal findings have also differed between adolescents and adults [19], and several comparable series have been limited to adults [4,13,20,21,22,23]. The remaining 603 examinations, one per patient, formed the study sample. The study was approved by the Non-Interventional Clinical Research Ethics Committee of Zonguldak Bülent Ecevit University before it began (approval no. 2024/23, dated 25 December 2024).

Prevalence of the CS and of its accessory canals was the primary outcome; the analyses involving age, sex, and side were secondary and exploratory. Sample size was planned around the pooled accessory canal prevalence of 54% given in the meta-analysis of de Oliveira-Neto et al. [24]. With *n* = *Z*^2^ × *p* × (1 − *p*)/*d*^2^, a 95% confidence level, and an absolute error of ±5%, 382 patients were required. Every eligible patient screened during the study period was included, so the final sample of 603 exceeded this number. The calculation was a planning step for the prevalence estimate, not a power calculation for the secondary analyses, which are therefore hypothesis-generating.

The study was designed and reported according to the STROBE checklist for cross-sectional studies; the completed checklist is given in Supplementary Table S1 [25].

Inclusion criteria:

1. Individuals aged 18 years or older.
2. Complete imaging of the anterior maxillary region, allowing assessment of the canalis sinuosus and accessory canals.
3. CBCT images of sufficient quality for diagnostic evaluation.
4. Images acquired according to the standard imaging protocol and technical parameters applied at the institution.

Exclusion criteria:

1. Image quality not suitable for diagnostic evaluation.
2. Records in which motion artifacts, metal-related streak or beam-hardening artifacts, or other artifacts [26] obscured the course of the canalis sinuosus or the alveolar and palatal bone in which the accessory canals run and open, so that these structures could not be assessed reliably.
3. Scans in which the anterior maxillary region required for the bilateral assessment of the canalis sinuosus and accessory canals did not lie entirely within the field of view, including scans whose coverage was partial or unilateral.
4. Presence of cystic, tumoral, inflammatory, or other pathological lesions that could alter the course of the canalis sinuosus and accessory canals.
5. Cases with supernumerary or impacted teeth in the anterior maxilla that could affect assessment of the canalis sinuosus or accessory canals.
6. Patients with a history of maxillofacial trauma that could affect the anatomy of the anterior maxilla.
7. Cases with a history of surgery that could alter the anatomical integrity of the anterior maxillary region.
8. Records in which the tooth-referenced terminal localization classification could not be applied because of missing incisors, canines, or first premolars in the anterior maxilla.

Figure 1 summarizes the selection of records and the steps of the radiological assessment.

### 2.2. Image Acquisition

All examinations were taken on a Veraviewepocs 3D R100/F40 unit (J. Morita Mfg. Corp., Kyoto, Japan) in our department, with an 8 cm × 10 cm field of view (FOV), 90 kVp, 5 mA, and a voxel size of 0.125 mm. Only examinations that covered the anterior maxilla completely and were of diagnostic quality entered the study.

Images were read in multiplanar mode with i-Dixel 2.0 software (J. Morita Corporation, Osaka, Japan). Axial, sagittal, and coronal sections were viewed together, and brightness, contrast, and magnification were adjusted whenever this helped to follow a canal. Measurements were made at the smallest slice thickness the unit and the software allowed.

Reading took place in a dimly lit room on a single monitor throughout: a 21.5-inch IPS panel with a resolution of 1920 × 1080 pixels (ASUS ZenScreen MB229CF, ASUS, Taipei, Taiwan).

### 2.3. Image Analysis and Measurements

Each volume was reoriented before measurement. In the sagittal plane the hard palate was set parallel to the horizontal reference line, in the axial plane the midsagittal line was aligned with the midline, and in the coronal plane the nasal floor was set parallel to the horizontal reference line.

For the CS we recorded presence, side, diameter, and the distribution of these findings by age and sex. For the accessory canals we recorded presence, side and localization, the number of canals per patient, diameter, morphological configuration, and the region in which each canal ended.

Two oral and maxillofacial radiologists, with more than five and more than three years of experience, read the images independently. The volumes were anonymized beforehand, and neither observer knew the reason for referral, the indication for the examination, or the patient’s demographic data; nor did they see each other’s records while reading. Both read every examination in the axial, coronal, and sagittal planes. Disagreements were settled jointly, once the independent readings and the reliability analyses had been completed, without recourse to a third observer. Whether the observers were aware of the numerical agreement statistics at that time was not recorded; because these statistics were calculated from the independent readings only, the consensus could not affect them. Diameters entered the analysis as the mean of the two readings.

Interobserver agreement was computed from the first, pre-consensus readings of all 603 examinations. For intraobserver agreement the primary observer re-read 91 randomly selected examinations after two weeks. Continuous variables were assessed with the intraclass correlation coefficient (ICC) from a two-way mixed-effects model with absolute agreement. Since the final measurements were the mean of two readings, the interobserver ICC is reported for average measures (*k* = 2), while the intraobserver ICC, which reflects the repeatability of one observer, is reported for single measures. Cohen’s kappa was used for the categorical variables (presence of a CS or accessory canal, localization, morphological type); for the presence variables it was calculated at side level (603 patients, 1206 sides), and for the localization and course of the accessory canals at canal level, each canal identified by both observers being treated as a separate unit and compared on the basis of the two independent, pre-consensus ratings. The 95% confidence intervals of all kappa coefficients accounted for within-patient clustering by resampling patients (2000 bootstrap samples). ICC values were interpreted following Koo and Li [27] (0.75–0.90, good; >0.90, excellent), and kappa values following Landis and Koch [28], with 0.81–1.00 taken as almost perfect agreement. Repeat readings were available for the primary observer only.

### 2.4. Assessment of the CS and ACs

**Figure 2:**
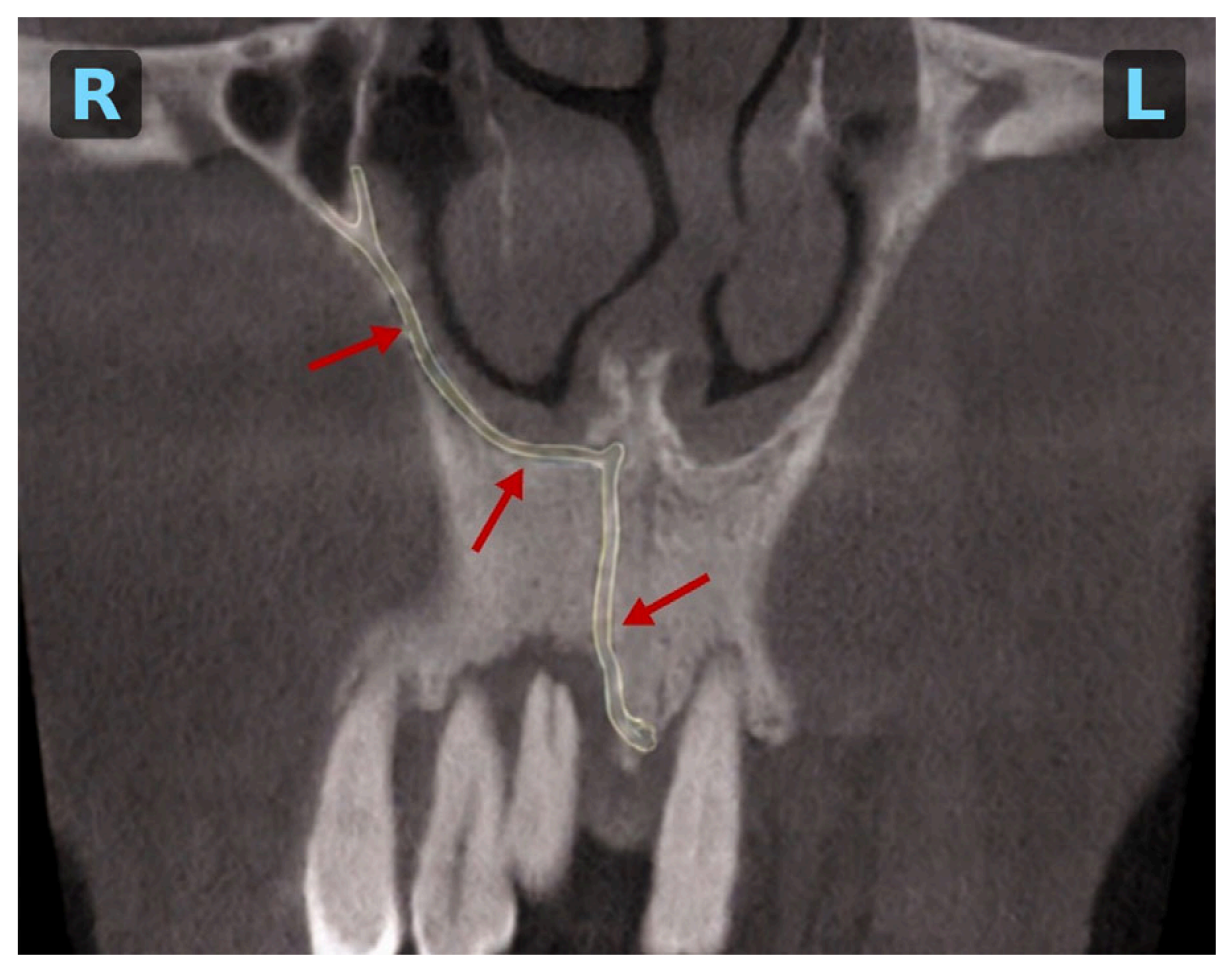
Coronal CBCT section showing the course of the canalis sinuosus (CS). Red arrows mark the canal, and the pale yellow contour highlights its traced course along the lateral wall of the nasal cavity toward the anterior alveolar process. The nasal cavity occupies the upper center of the image, and the maxillary anterior teeth lie at the bottom. R and L (blue) mark the patient’s right and left sides.

- Every examination was assessed in the axial, coronal, and sagittal planes.
- A canal was accepted as the CS when it could be followed on multiplanar sections along its typical route, from the infraorbital canal along the lateral wall of the nasal cavity to the anterior maxilla [29,30,31,32], and measured at least 0.5 mm; sides meeting both conditions were recorded as CS-positive (Figure 2).
- The right and left sides of the maxilla were analyzed separately.
- The same 0.5 mm limit was applied to the accessory canals, using the mean of their two perpendicular diameters (Section 2.4.3). A canal of 0.5 mm spans four voxels at the voxel size used (0.125 mm), but the effective spatial resolution of clinical CBCT is coarser than its voxel size, and structures of about this size lie close to the practical limit of discrimination [33]. The same limit has been used in comparable studies [13,19,20,21,29]. Canals below this limit were not recorded, so the prevalences reported here describe what can be detected on CBCT under these criteria rather than what is anatomically present.

#### 2.4.1. CS Diameter Measurement

- The measurement was made on the segment that runs vertically beside the nasal aperture.
- It was taken at the widest point of the canal [29].

The widest point was used because the cross-section of the canal varies along this segment and its widest point can be located reproducibly by scrolling through consecutive sections; the same reference was used by Beckenstrater et al. [29].

#### 2.4.2. Definition and Measurement of the ACs

**Figure 3:**
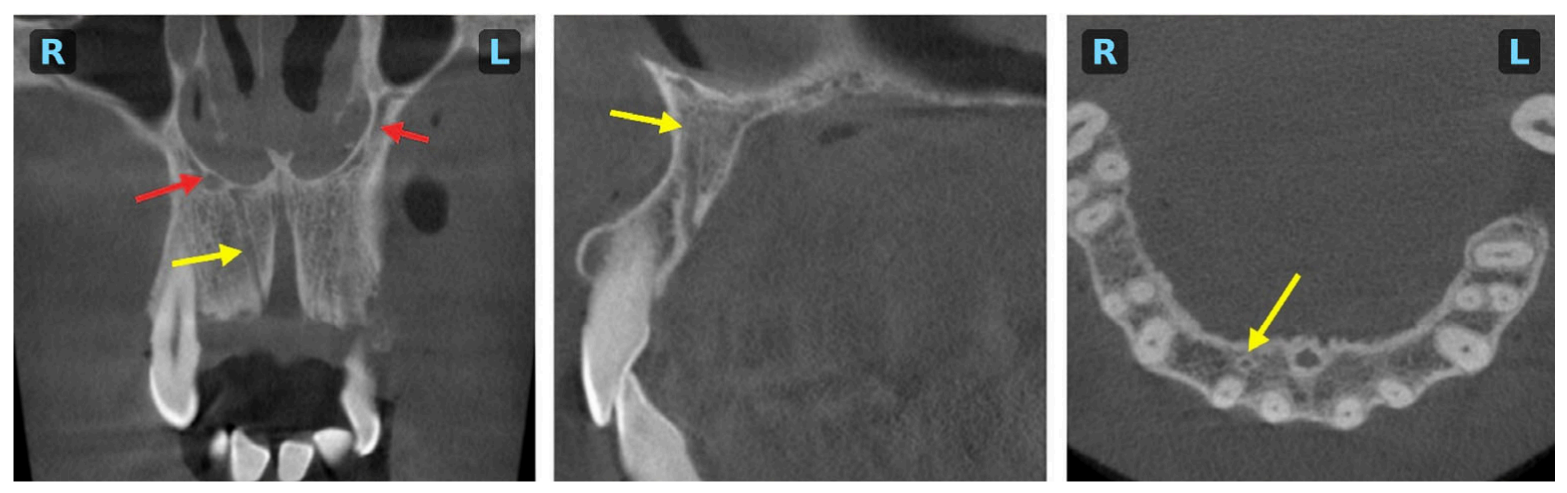
Accessory canal (AC) and canalis sinuosus (CS) on coronal (**left**), sagittal (**middle**), and axial (**right**) CBCT sections. Red arrows mark the CS on both sides of the nasal cavity in the coronal section; yellow arrows mark the AC in the coronal and sagittal sections and its cross-section in the anterior maxilla in the axial section. R and L (blue) mark the patient’s right and left sides in the coronal and axial sections.

An accessory canal was defined as a secondary canal of at least 0.5 mm whose continuity with the CS could be demonstrated on multiplanar sections. Two morphological types met this definition (Figure 3):

- Canals continuing from the terminal end of the CS.
- Secondary canals branching from the main canal.

Canals whose connection with the CS could not be demonstrated were left out. This rule was adopted because the anterior maxilla also contains bone canals that do not communicate with the CS; in the series of von Arx et al., only 56.7% of the accessory canals did [34]. Counting them would have mixed CS branches with other neurovascular canals, but the rule also means that branches whose connection was interrupted on the images were not counted, which makes the accessory canal figures conservative. Each accessory canal was counted separately and followed from the point where it left or continued the CS to the foramen through which it opened onto the maxillary surface; one terminal localization and one course type were recorded for each.

#### 2.4.3. Assessment of AC Diameter

- Both measurements were made on the same axial section, chosen as the section on which the borders of the canal lumen could be distinguished most clearly.
- The canal was measured in two perpendicular directions.
- The mean of the two values was taken as the diameter.

Measuring in two directions limits the error introduced by the oval cross-section of the canal and follows the approach of Yeap et al. [3]. The 0.5 mm threshold was applied to this mean value, and the agreement between the two diameters was examined to show how far the canals departed from a circular cross-section (Section 2.5).

#### 2.4.4. Terminal Point of the ACs

**Figure 4:**
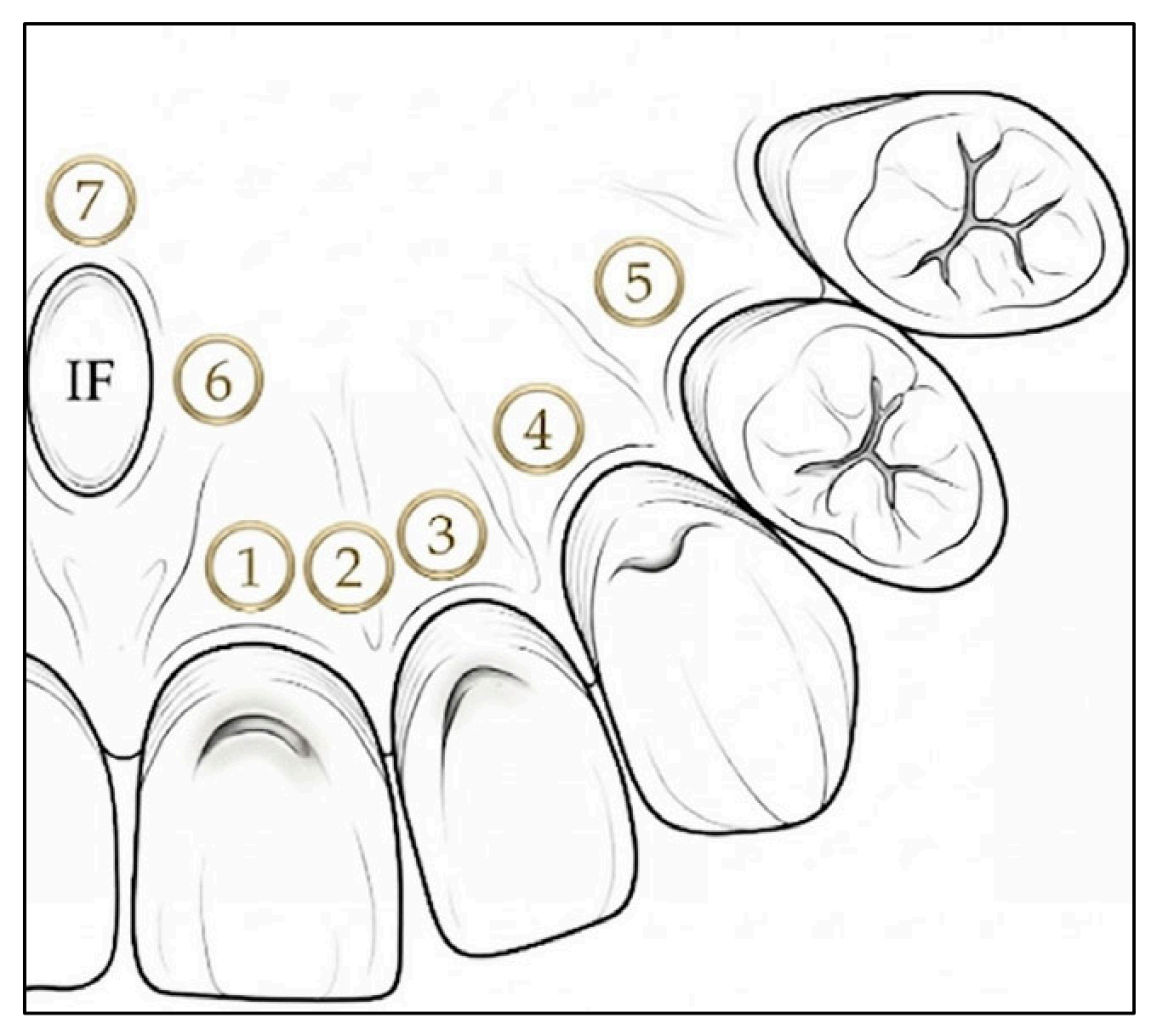
Schematic illustration of the palatal localization classification used for the terminal points of the accessory canals, based on the classification system proposed by de Oliveira-Santos et al. [35]. One half of the anterior maxilla is shown from the palatal aspect, with the midline at the left edge of the drawing. The numbered circles mark the seven regions: 1, central incisor region; 2, between the central and lateral incisors; 3, lateral incisor region; 4, canine region; 5, first premolar region; 6, lateral to the incisive foramen; 7, posterior to the incisive foramen. IF, incisive foramen. The figure was created by the authors for illustrative purposes; the ‘other localization’ category is not included in the drawing.

de Oliveira-Santos et al. [35] proposed a scheme for describing where the canalis sinuosus and its accessory canals open onto the palate (Figure 4). It divides the region into seven areas, defined by their relation to the teeth and to the incisive foramen:

1. Central incisor region.
2. Area between the central and lateral incisors.
3. Lateral incisor region.
4. Canine region.
5. First premolar region.
6. Region lateral to the incisive foramen.
7. Area posterior to the incisive foramen.

We used this seven-region scheme; canals that ended outside these areas were grouped as ‘other localization’. The foramen through which a canal opened onto the maxillary surface was identified on axial sections and located with reference to these landmarks [34,35,36].

#### 2.4.5. Classification of the ACs According to Course

**Figure 5:**
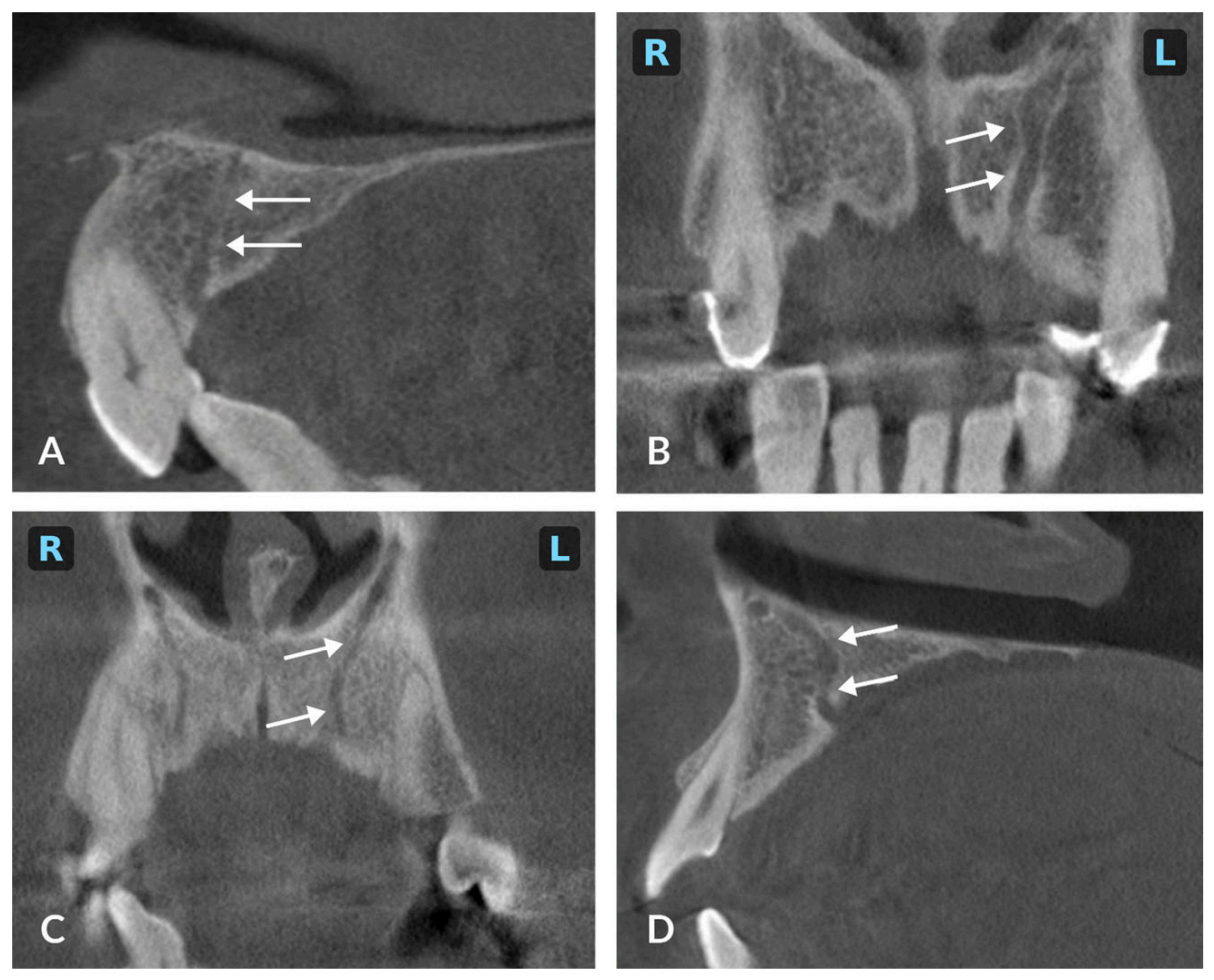
Course configurations of the accessory canals (ACs) of the canalis sinuosus: (**A**) straight vertical; (**B**) curved medial; (**C**) curved lateral; (**D**) curved distal. White arrows mark the AC in each panel. Panels (**A**,**D**) are sagittal sections, with the incisor crowns at the lower left and the palate extending to the right; panels (**B**,**C**) are coronal sections, with the nasal cavity at the top and the maxillary teeth at the bottom; R and L (blue) mark the patient’s right and left sides.

The course of each accessory canal was judged qualitatively, using the morphological descriptions available in the literature [5,34], and grouped by its orientation within the bone (Figure 5):

- Canals running straight in a vertical direction.
- Canals angled toward the midline (curved medially).
- Canals deviating laterally.
- Canals running in a distal direction.

### 2.5. Statistical Analysis

Analyses were carried out in Python 3.12.13 (Python Software Foundation, Wilmington, DE, USA) with pandas 2.2.3, NumPy 2.3.5, SciPy 1.17.0, and statsmodels, including the agreement statistics; the analyses added during peer review were run in Python 3.13.16 with pandas 3.0.5, NumPy 2.5.3, SciPy 1.18.1, and statsmodels 0.15.0.

Continuous variables are given as mean ± standard deviation and as median with interquartile range, categorical variables as counts and percentages. Confidence intervals for the prevalence figures were obtained by the Wilson method. Age was grouped into five categories for the descriptive tables (≤29, 30–39, 40–49, 50–59, and ≥60 years) and kept continuous in the models. At patient level, the presence of a CS or an accessory canal was compared between sexes with Fisher’s exact test and across age groups with Pearson’s chi-square test. A linear trend across the five ordered age groups was examined with the Cochran–Armitage test with equally spaced scores, together with a chi-square test for departure from linearity [37].

Three units of analysis were used, and each table states its denominator. Prevalence is reported per patient (*n* = 603), a canal on either side counting as present; side comparisons and the presence models use sides (1206 sides, two per patient); CS diameter uses the CS-positive sides (634 sides in 321 patients); and accessory canal diameter, terminal localization, and course use individual canals (385 canals in 191 patients). Where more than one observation came from the same patient, the models treated the patient as a cluster; the canal-level distributions of localization and course were not tested and are reported descriptively. Percentages are given to one decimal place, diameters in millimeters to two decimal places (differences and regression coefficients to three), and *p* values to three decimal places.

Right and left sides were compared with the exact McNemar test, which uses only the discordant patients, those with a canal on one side only. It therefore tests the symmetry of presence between the two sides, that is, whether right-only and left-only cases are equally frequent, rather than comparing two independent prevalences; patients with canals on both sides or on neither side do not contribute to it. In patients with a bilateral CS, the difference in diameter between sides was tested with a paired *t* test and, as a sensitivity analysis, with the Wilcoxon signed-rank test.

Side-level presence of the CS and of accessory canals was modeled with generalized estimating equations (GEE), using a binomial distribution and a logit link, with patient as the cluster, an independence working correlation, and robust (sandwich) standard errors. Age entered as a linear and a quadratic term, centered and scaled per 10 years, since its relationship with canal presence need not be linear; the overall effect of age was tested with a joint two-degree-of-freedom Wald test. Diameters were modeled with GEE using a Gaussian distribution and an identity link, pairing sides for the CS and clustering canals within patients for the accessory canals, with age as a linear term. Age, sex, and side were entered together in every model (reference: female sex, right side). Binary outcomes are reported as odds ratios and continuous outcomes as adjusted beta coefficients, both with 95% confidence intervals; age effects are also shown as standardized marginal probabilities. GEE was preferred to generalized linear mixed models because the question was how prevalence varies across patients in the population, which GEE estimates directly as population-averaged effects, whereas a random-intercept model estimates effects conditional on the individual patient [38,39]. With only two sides per patient, a random-intercept model also becomes unstable when the two sides are almost always concordant, as they were for the CS.

Three families of comparisons were defined for multiplicity control: the four subgroup comparisons of CS and accessory canal presence by sex and age group; the eight age and sex terms of the four GEE models (CS presence, AC presence, CS diameter, AC diameter); and the four side terms of the same models. The false discovery rate was controlled within each family by the Benjamini–Hochberg procedure, and raw *p* values are given alongside adjusted *q* values. As a check on the side-level models, patient-level logistic regressions with robust standard errors were fitted with the presence of any CS and of any accessory canal as outcomes and the same age and sex terms (Supplementary Table S2).

Five further analyses were added during peer review; they are reported with unadjusted *p* values and were not included in the three families: the Cochran–Armitage trend test described above; random-intercept logistic models of side-level canal presence, fitted by maximum likelihood with Gauss–Hermite quadrature, as a check on the GEE models; a patient-level model of accessory canal presence restricted to CS-positive patients, to separate the age association of the accessory canals from that of the CS; the agreement between the two perpendicular accessory canal diameters, assessed as their mean difference with a cluster-robust confidence interval, Bland–Altman limits of agreement [40], and a two-way, absolute-agreement, single-measures ICC with a patient-level bootstrap confidence interval (2000 resamples); and the accessory canal prevalence that would have resulted from stricter diameter thresholds (0.6, 0.7, 0.8, and 1.0 mm).

All tests were two-sided. Significance was set at *p* < 0.05 where no correction was applied and at *q* < 0.05 within the three families.

## 3. Results

The 603 patients had a mean age of 46.2 ± 14.7 years (median 48.0 years; IQR 36.5–57.0; range 18–83 years); 317 (52.6%; 95% CI 48.6–56.5) were men and 286 (47.4%; 95% CI 43.5–51.4) women. Table 1 gives the demographic data together with the main prevalence figures. Table 1 and Table 2 give patient-level prevalence, Table 3 the diameters, Table 4 the terminal localization and course of individual canals, and Table 5 the adjusted models.

### 3.1. Observer Agreement

Interobserver agreement was calculated from the two independent readings of all 603 examinations, before consensus. Cohen’s kappa was 0.91 (95% CI 0.88–0.94) for the presence of a CS, 0.88 (95% CI 0.84–0.92) for an accessory canal, 0.84 (95% CI 0.80–0.88) for its localization, and 0.82 (95% CI 0.77–0.87) for its course. Average-measures ICC was 0.97 (95% CI 0.96–0.97) for CS diameter and 0.92 (95% CI 0.91–0.94) for accessory canal diameter.

In the 91 examinations re-read by the primary observer, kappa was 0.95 (95% CI 0.90–0.98) for the presence of a CS, 0.92 (95% CI 0.84–0.97) for an accessory canal, 0.90 (95% CI 0.81–0.95) for localization, and 0.88 (95% CI 0.77–0.94) for course; single-measure ICC was 0.97 (95% CI 0.95–0.98) for CS diameter and 0.91 (95% CI 0.87–0.94) for accessory canal diameter. Cases on which the observers disagreed were resolved by consensus once these analyses were complete.

### 3.2. Prevalence of the Canalis Sinuosus and Accessory Canals

The CS was found in 53.2% of the patients and was bilateral in 313 of these 321 patients (97.5%), that is, in 51.9% of the whole sample (Table 1). Only eight patients had a unilateral CS, three on the right and five on the left, and this difference was not statistically significant (exact McNemar test, *p* = 0.727).

Accessory canals were less common, affecting 31.7% of the patients, and were bilateral in 83 of the 191 AC-positive patients (43.5%; 13.8% of the whole sample) (Table 1). Of the 108 patients with an accessory canal on one side only, 46 had it on the right and 62 on the left, a difference that was not statistically significant (exact McNemar test, *p* = 0.149). Patients with accessory canals had 2.0 ± 1.2 of them on average, 385 in all. Since an accessory canal had to be continuous with the CS, every AC-positive patient was also CS-positive, and a CS-negative side could not carry an accessory canal. Among CS-positive patients, 59.5% had at least one accessory canal (191/321; 95% CI 54.0–64.7); per side the figure was 40.8% on the right (129/316; 95% CI 35.5–46.3) and 45.6% on the left (145/318; 95% CI 40.2–51.1).

No statistically significant difference in the prevalence of either canal was found between men and women (Table 2). Both were least common in the 30–39 age group and most common in the 50–59 group, but none of the four comparisons survived Benjamini–Hochberg correction (all *q* ≥ 0.113). The post hoc Cochran–Armitage test indicated a linear trend toward higher prevalence across the ordered age groups for both the CS (χ^2^(1) = 5.70; *p* = 0.017) and the accessory canals (χ^2^(1) = 4.51; *p* = 0.034), without significant departure from linearity (*p* = 0.321 and *p* = 0.140); the confidence intervals of adjacent age groups overlapped widely (Table 2).

### 3.3. Diameter Measurements of the Canalis Sinuosus and Accessory Canals

The two sides gave almost the same CS diameter (Table 3), and in patients with a bilateral canal the difference was not significant (paired *t* test, *p* = 0.754; Wilcoxon signed-rank test, *p* = 0.495).

CS diameter was clearly larger in men (Table 3); the adjusted, cluster-robust estimate of this difference is given in Table 5.

Accessory canals were much narrower than the main canal, with similar values on the two sides (Table 3).

The two perpendicular diameters of the accessory canals showed no systematic difference (Supplementary Table S3). Their mean difference was −0.007 mm (95% CI −0.018 to 0.005), the 95% limits of agreement were −0.25 to 0.23 mm, and the ICC was 0.79 (95% CI 0.75–0.83). The two values differed by no more than one voxel (0.125 mm) in 278 canals (72.2%) and by no more than two voxels in 374 (97.1%); the larger diameter exceeded the smaller by a median of 11% (IQR 7–18%).

The distribution of accessory canal diameters peaked at 0.60–0.69 mm, with 102 canals (26.5%), whereas only 19 (4.9%) measured 0.50–0.59 mm. Raising the threshold reduced the accessory canal prevalence from 31.7% at 0.5 mm to 30.2% at 0.6 mm, 24.2% at 0.7 mm, 17.6% at 0.8 mm, and 5.8% at 1.0 mm (Supplementary Table S4). No CS-positive side measured less than 0.83 mm.

### 3.4. Localization and Course of the Accessory Canals

In this section, percentages refer to the 385 canals rather than to patients. Most accessory canals ended in the lateral incisor region (26.5%), followed by the central incisor region (22.9%); the area between the central and lateral incisors and the canine region were equally frequent (16.9% each). The first premolar region was the rarest of the defined areas (2.6%; Table 4). Right and left sides gave broadly similar distributions. Taken together, 320 canals (83.1%) ended between the central incisor and canine regions. At patient level, 120 patients (19.9% of the sample; 62.8% of AC-positive patients) had at least one accessory canal ending in the lateral incisor or canine region.

Most canals followed a straight vertical route, and the remainder curved medially, laterally, or distally (Table 4). A distal curve, and a termination in the first premolar region, were both more frequent on the left in absolute numbers; these side differences were not tested and should be read as descriptive only.

### 3.5. Adjusted (GEE) Analyses of Age, Sex and Side

**Figure 6:**
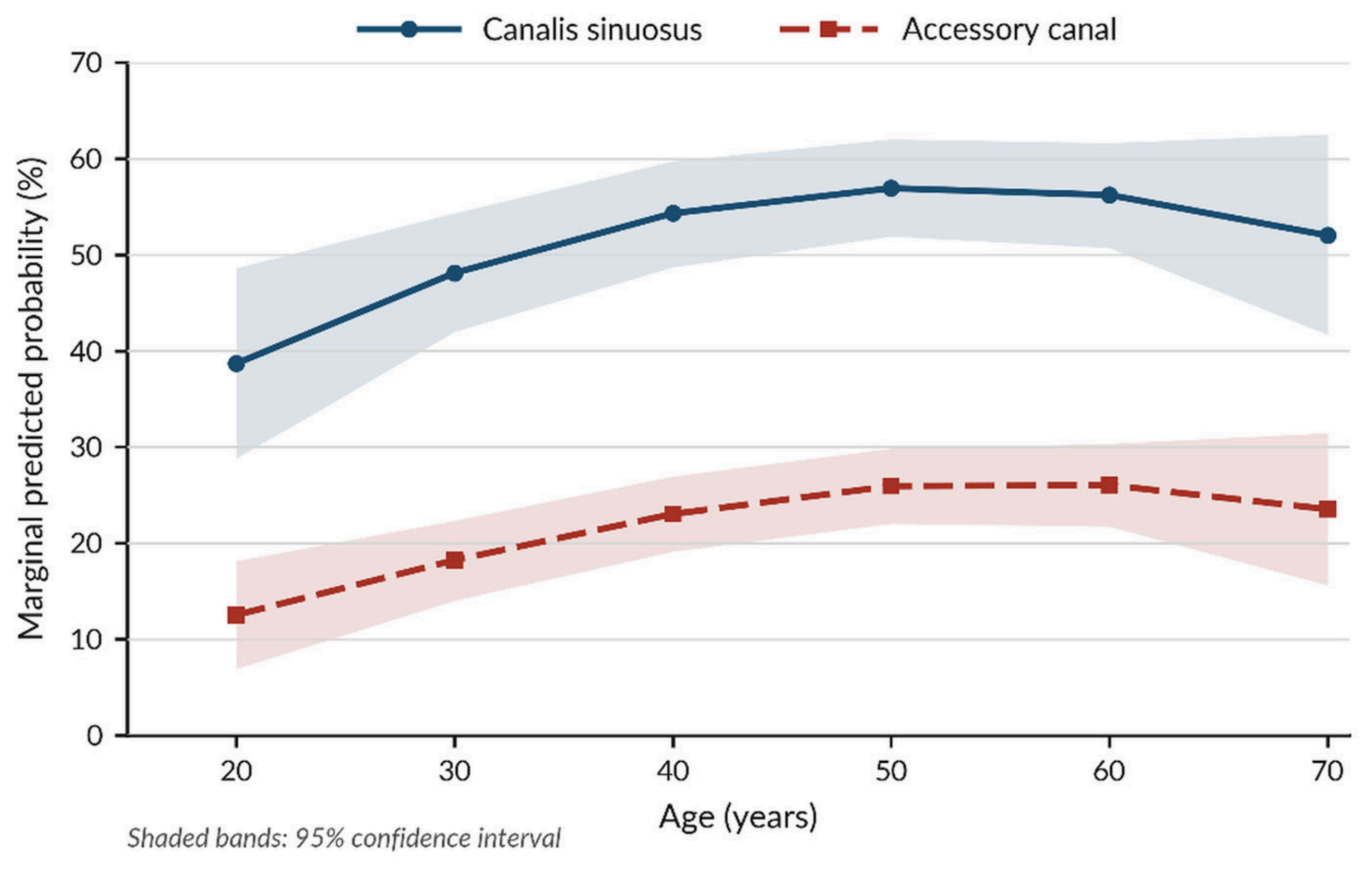
Marginal predicted probabilities of canalis sinuosus (CS, solid line with circles) and accessory canal (AC, dashed line with squares) presence across age, derived from the GEE models in Table 5. The horizontal axis shows age in years and the vertical axis the predicted probability (%) that a canal is present on a given side. Markers show the estimates at 10-year intervals, standardized over the observed distribution of sex and side, and the shaded bands show their 95% confidence intervals. Age was modeled with linear and quadratic terms. Probabilities are estimated at the side level (1206 sides from 603 patients) and are therefore lower than the patient-level crude prevalences in Table 2. Curves are displayed over the 20–70-year range, where the data are densest.

In the cluster-robust GEE models, the linear and quadratic age terms were jointly significant for the presence of the CS (χ^2^(2) = 8.41; *p* = 0.015; *q* = 0.040) and for the presence of accessory canals (χ^2^(2) = 8.98; *p* = 0.011; *q* = 0.040). The marginal predicted probabilities rose from young adulthood into middle age, reached a plateau in the fifties, and then fell slightly (Figure 6). This curvature should be read descriptively: the confidence intervals widen considerably at the upper end of the age range, and across the age groups the departure from a linear trend was not significant (Table 2). The direction of this pattern matches the crude patient-level prevalences by age group in Table 2; the absolute values are lower because the probabilities are estimated per side rather than per patient.

No statistically significant association of sex or side with the presence of either canal was found (Table 5).

For the continuous outcomes, the CS was wider in men than in women (*β* = 0.227 mm; 95% CI 0.183–0.271; *p* < 0.001; *q* < 0.001), while no statistically significant association of its diameter with age or side was found. Accessory canal diameter showed no statistically significant association with age, sex, or side. Table 5 lists the effect estimates, confidence intervals, and Benjamini–Hochberg-adjusted *q* values for all four models.

Repeating the analysis at patient level, with logistic regression in place of the side-level GEE models, left the joint age effect significant for both the CS (*p* = 0.022) and the accessory canals (*p* = 0.026), and the sex effects nonsignificant (CS: OR 1.01, 95% CI 0.73–1.41; accessory canals: OR 1.19, 95% CI 0.83–1.69; Supplementary Table S2). A random-intercept logistic model of accessory canal presence led to the same conclusions (joint age effect *p* = 0.011; male sex OR 1.36, 95% CI 0.70–2.63; left side OR 1.35, 95% CI 0.92–1.97). For CS presence, the fit of such a model was degenerate: because the two sides were concordant in 595 of the 603 patients, the between-patient standard deviation was estimated at about 60 on the logit scale, and the subject-specific estimates were uninformative. Among the 321 CS-positive patients, accessory canal presence showed no statistically significant association with age (joint *p* = 0.321) or sex (OR 1.32, 95% CI 0.83–2.09) in a post hoc model (Supplementary Table S2).

## 4. Discussion

We examined the canalis sinuosus and its accessory canals in 603 CBCT examinations, recording prevalence, side distribution, diameter, terminal localization, and course together in the same sample. The CS was present in 53.2% of patients, bilaterally in 97.5% of those. Accessory canals were found in 31.7% of the whole sample and in 59.5% of CS-positive patients. No statistically significant side difference was found for either structure. The main canal was wider in men than in women, and in exploratory analyses age was associated with the presence of both canals. Accessory canals ended most often in the lateral incisor region and usually ran straight vertically. Because the sample size was planned for the prevalence estimates rather than for the demographic comparisons, these associations, and the absence of others, are best regarded as hypothesis-generating.

Our figure of 53.2% falls in the middle of a very wide range [1,11,15,30]. It is close to the 59.75% of Rao et al. [41], although their figure referred to CS openings on the palatal side of the alveolar ridge, and far below the near-universal prevalence of Tetik and Zafersoy Akarslan [21], who saw a bilateral CS in all 568 of their patients. At the other extreme, Allaberdiyev et al. [42] reported 18.4% in a Turkish sample, about a third of our value; their images were screened for a long list of anatomical structures before implant placement rather than for the CS in particular, which may well explain the difference. The systematic review of de Oliveira-Neto et al. [24] shows how persistent this heterogeneity is. Population differences alone cannot account for it: the spatial resolution of the scanner, the voxel size, the segment of the canal examined, and the criteria used to call a canal present all contribute [1,3,34]. The comparison is closer when the threshold is held constant (Table 6). In the series that applied the same 0.5 mm limit, the CS was recorded in virtually every patient [13,19,21,29], although most of them used larger voxels than ours, so the lower figure in our sample is unlikely to reflect resolution. We cannot fully explain the difference. Our requirement that the canal be followed along its whole route from the infraorbital canal to the anterior maxilla may have been applied more strictly than elsewhere, and observers may differ in how they judge a faint, partly corticated canal. Our figure should therefore be read as the prevalence of a CS that could be traced under our criteria, not as an anatomical prevalence, and failure to trace the canal does not mean that the anterior superior alveolar neurovascular bundle is absent [2,14,32].

That 97.5% of our CS-positive patients had the canal on both sides, without a statistically significant side difference, indicates that the CS is usually a symmetrical structure. Published bilaterality rates nevertheless vary widely [11,15]. How far distally the canal is followed, and whether its distal continuation is counted as part of the main canal or as a separate accessory branch, seem to us the most likely sources of that variation.

The overall accessory canal prevalence of 31.7% is close to the 34.7% of Tomrukçu and Köse [5], who recorded canals of any size, and the 35.5% of Tercanli Alkis et al. [13], who used the same 0.5 mm limit, but other Turkish series have reported figures from 2.2% of the whole sample, with a CS detected in only 8.9% of patients [15], to 70.8% for canals of about 1 mm [43]. Our requirement of demonstrable continuity with the CS is stricter than definitions that counted any bone canal in the region, and it probably contributes to our lower overall figure: in the series of von Arx et al., only 56.7% of the accessory canals communicated with the CS [34]. The figure depends directly on how often the CS itself is detected, since an accessory canal must by definition continue from it. Gonzales-Campbell et al. [44] reported 62.1% in a Latin American sample in which every patient had a CS, and Beckenstrater et al. [29] reported 58.8% with a CS detection rate of 99.6%; both figures therefore describe accessory canal frequency among CS-positive individuals. Ours, 59.5%, is very close to theirs, which suggests that part of the apparent difference in overall prevalence reflects CS detection rather than the populations studied. Among the series in which the CS was found in virtually every patient, however, accessory canal prevalence still ranged from 35.5% to 67.6% at the same 0.5 mm limit (Table 6), so CS detection is not the whole explanation. The diameter threshold matters just as much: within a single sample, Wu et al. [20] found accessory canals in 80.4% of patients at ≥0.5 mm and in 51.7% at ≥1 mm. Comparisons between studies are meaningful only when the threshold, the definition of an accessory canal, and the CS detection rate are read together.

The 0.5 mm limit deserves separate comment, because it shapes both the prevalence and the diameters reported here. At a voxel size of 0.125 mm, a canal of 0.5 mm spans about four voxels, but the effective resolution of a clinical CBCT scan is coarser than its voxel size: in scans of living patients a resolution of only just above 1 line pair per millimeter can be expected [33], which places structures of about 0.5 mm near the limit of what can be separated from their surroundings. Close to this limit, partial-volume averaging blurs the thin cortical wall of a canal into the neighboring bone [26], so that fine canals may be missed and the diameter of those detected may be misjudged by up to a voxel at each margin, an error that is proportionally much larger for a canal of 0.6 mm than for a CS of 1.3 mm; how well fine structures are depicted depends on the voxel size and on the diagnostic task [45]. Our data are consistent with this. Accessory canals of 0.60–0.69 mm were common (26.5% of all canals), but those of 0.50–0.59 mm were scarce (4.9%), a sharp fall just above the threshold that fits declining detectability better than a genuine absence of finer canals. The accessory canal figures are therefore lower bounds, and they depend steeply on the threshold: in our own sample, prevalence fell from 31.7% at 0.5 mm to 5.8% at 1.0 mm (Supplementary Table S4). Wu et al. found a change in the same direction between these two limits [20], but a smaller one, possibly because they measured the canals at their openings, whereas we measured them on an axial section of the canal. The CS was much less affected; no CS-positive side measured less than 0.83 mm, so CS detection depended mainly on whether the canal could be traced continuously. That continuity rule excluded channels that could not be traced back to the CS and made the classification reproducible, but it will also have excluded true branches whose connection was obscured. Both rules therefore bias the prevalence estimates downward, and studies that used a 1 mm limit or recorded any bone canal are not directly comparable with ours.

We found no statistically significant association between sex and the presence of either canal, as in the series of Gonzales-Campbell et al. [44] and Eberliköse et al. [46]. The CS was nonetheless wider in men, which agrees with Baliutavičiūtė et al. [22] but not with Aktuna Belgin et al. [15], who found no sex difference in diameter. Sex may therefore be related to selected morphometric properties rather than to whether a canal is present, although the study was not powered to exclude modest differences in prevalence; accessory canal diameter, for instance, showed no sex difference in our data. Where along the canal the measurement is taken may matter as much as the population studied. We measured the CS at its widest point on the vertical segment beside the nasal aperture, as Beckenstrater et al. did [29]; diameters taken at fixed levels, such as the junction with an accessory canal [13] or the piriform aperture [19], measure a different quantity, and mean diameters should not be compared without also comparing measurement protocols. Samunahmetoglu and Kurt [47] reported a mean CS diameter of about 1.3 mm, close to ours, which suggests that this measurement is reasonably stable across populations.

Accessory canals ended most often in the lateral incisor region (26.5%), in keeping with earlier work [4,7,13,35]; Valizadeh et al. [23] also found this region to be the most common end point. The dominant region is not the same in every series: Ghandourah et al. [19] found most canals in the central incisor region, and Beckenstrater et al. [29] reported that 57.2% ended in the anterior palatal region. Such comparisons depend on the diameter threshold as much as on the regional definitions, and de Oliveira-Santos et al. [35], whose scheme we used, recorded only canals of ≥1 mm. In our sample the central incisor, lateral incisor, and canine regions, together with the area between the central and lateral incisors, accounted for 83.1% (320 of 385) of end points, which places these canals in the area used for implants, apical surgery, and other anterior maxillary procedures. The straight vertical course of most canals (63.4%) matches the vertical orientation reported by Eberliköse et al. [46].

Age behaved differently depending on how it was handled. Grouped into categories, prevalence peaked in the 50–59 group, but no comparison survived correction for multiplicity; modeled continuously with a linear and a quadratic term, age had a significant overall effect on both canals (*q* = 0.040). Three differences between the analyses explain this. The chi-square test is an omnibus test with four degrees of freedom that ignores the order of the age groups, whereas the GEE models spend two degrees of freedom on a smooth age effect and use each patient’s exact age; categorization discards information and costs power, particularly when the underlying relationship is curved. The raw *p* values were close to 0.05 in both analyses (0.056 and 0.040 for the age-group tests; 0.015 and 0.011 for the GEE terms), so the outcome of the Benjamini–Hochberg procedure depended on the rank of each *p* value within its family: among the four subgroup comparisons the two age tests ranked first and second (*q* = 0.113), whereas among the eight GEE terms they ranked second and third, behind the sex effect on CS diameter (*q* = 0.040). Finally, a test that respects the order of the age groups showed a linear trend for both canals (*p* = 0.017 and *p* = 0.034), so the categorical data point in the same direction as the models. A peak in the fifties is consistent with Schnutenhaus et al. [48], who found accessory foramina concentrated in the fourth to sixth decades. The joint Wald test, however, asks whether age matters at all, not whether the relationship departs from linearity, and a quadratic term forces a single peak onto the curve; the position of that peak in Figure 6 and the size of the later decline should therefore be read descriptively, all the more because the departure from a linear trend across the age groups was not significant. The age effect on accessory canals may also be partly an effect on the CS, since one cannot be recorded without the other; among CS-positive patients, accessory canal presence showed no statistically significant association with age (*p* = 0.321), which is compatible with this interpretation, although the crude proportions in this subgroup still rose and fell across the age groups. The sample size was not planned for these comparisons, and the exclusion of patients with missing anterior teeth, who are likely to be older, may have made the oldest group a selected one, so the age findings are hypothesis-generating. In a cross-sectional sample, none of this should be taken to mean that canals form or disappear as a person ages.

Prevalence figures for the CS and its accessory canals will probably continue to vary between series, largely for methodological reasons. The location of the accessory canals appears more consistent, as they cluster in the anterior dentoalveolar region in one study after another, whereas the evidence on sex differences remains mixed [14,15,16,17,22,44,46].

### 4.1. Clinical Implications

Whatever its exact prevalence, the CS matters clinically because of what it carries and where it lies [7,31,35]. Yılmaz et al. [49] showed how variable the position and size of maxillary neurovascular structures can be and argued for three-dimensional assessment before surgery, and pain, neurosensory disturbance, and bleeding have all been reported after the canal or its branches were damaged during implant placement [48]. In published cases, symptoms resolved after the implant was replaced with a shorter one or removed [50,51], and chronic neurosensory disturbance has been traced to invasion of the CS on postoperative CBCT [52]. We did not record surgical outcomes, so our data cannot establish that a particular diameter or location raises the risk of complications, nor do they justify taking a CBCT for the sole purpose of looking for these canals. What they do support is a systematic look at the anterior maxilla whenever a CBCT has been taken for another valid reason. Table 7 separates what was observed in this study from the clinical inferences drawn from it; the inferences were not tested here.

The lateral incisor and canine sites deserve particular attention. Together they received 43.4% of the accessory canals, and 19.9% of the patients had at least one canal ending there; both are also common implant sites [9]. When CBCT is available for planning at these sites, the canal should be sought on the cross-sectional images palatal to the planned implant, from the nasal floor to the alveolar crest, bearing in mind that more than one canal may be present and that 141 of the 385 canals in our sample (36.6%) curved rather than running straight. No safety distance has been validated for the CS. A zone of at least 2 mm is advised around the inferior alveolar and mental nerves [53] and has been suggested for the CS [23], and the same margin is recommended for static guided implant surgery, whose mean deviation at the implant apex is 1.4 mm [54]. A 2 mm margin is therefore a reasonable working distance, but it is an extrapolation, not a result of this study. Where an accessory canal lies closer than this to the planned osteotomy, the position, angulation, or length of the implant can be changed; static guides or dynamic navigation, with mean apical deviations of about 1.0–1.4 mm [54,55], help to keep the planned distance but cannot protect a margin smaller than their own error; and a restoration that does not require an implant may sometimes be preferable [56]. Because canals narrower than about 0.5 mm were not detected, a negative CBCT does not exclude neurovascular tissue at the site, and unexplained neurosensory symptoms after surgery in this region justify re-evaluation with CBCT [52].

### 4.2. Generalizability and Differences Between Studies

The figures describe adults referred for CBCT to a single university clinic in the Western Black Sea region of Türkiye and examined on one scanner with one protocol. Referral for implant planning, impacted teeth, or suspected pathology is unlikely to select patients for the presence of a CS, but a clinical sample is not a random sample of the population, and the prevalences should not be read as population estimates. The exclusion criteria also shaped the sample. Patients with missing anterior teeth, previous surgery, trauma, or pathology in the region were excluded, so the results describe dentate anterior maxillae without previous intervention. Older patients, who are more likely to have lost anterior teeth, therefore form a selected group, which may have influenced the age findings; and because a missing tooth was the last reason in the exclusion hierarchy, the 27 records excluded for it may understate how many screened patients were missing these teeth. The findings cannot be transferred directly to edentulous sites, which are where implants are placed. The method could be adapted to such sites by locating each foramen relative to landmarks that persist after tooth loss, such as the incisive foramen, the midline, and the alveolar crest, rather than relative to teeth, as Schnutenhaus et al. did by using the alveolar cortex as the reference where no tooth was present [48]; whether canals at edentulous sites behave like those in dentate jaws remains to be shown. Differences between populations may well exist, but they cannot be separated from methodological ones at present: even the studies in Table 6, which share the same threshold, differ in voxel size, in the canals to which the threshold was applied, and in CS detection, and other series differ in the threshold itself, in the definition of presence, and in the segment measured. These differences would have to be standardized before population differences could be judged.

### 4.3. Strengths and Limitations

The sample was large, well above the number required by the sample size calculation, and every examination came from the same unit and the same protocol at a voxel size of 0.125 mm. Two observers read the images independently, and interobserver reliability was calculated before any consensus was reached, which gives a realistic picture of reproducibility. On the statistical side, the dependence between the two sides of a patient was modeled explicitly, the false discovery rate was controlled within predefined families of comparisons, and the side-level results were checked against patient-level models.

The study was retrospective and single-center. Its patients had been referred for clinical reasons, so the prevalences reported here are not population figures. The 0.5 mm threshold and the requirement of continuity with the CS made the assessment reproducible but will have excluded finer canals, and canals whose connection could not be traced; near the threshold, partial-volume effects also limit the precision of individual diameters. Patients missing an incisor, canine, or first premolar were left out so that the tooth-based localization scheme could be applied, which means the findings cannot be transferred directly to edentulous sites awaiting implants, and recording a single exclusion reason per record may have hidden co-existing reasons and introduced selection bias. The canals were identified on CBCT alone, without histological or cadaveric confirmation, and no surgical outcomes or complications were assessed, so the clinical implications discussed above are inferences from anatomy. The cross-sectional design, finally, rules out any conclusion about anatomical change with age.

The reliability assessment has limitations of its own. Only the primary observer repeated the readings, so the repeatability of the second observer was not quantified directly. The consensus classification of discordant cases may also carry bias, for example toward the judgment of the more experienced observer; whether the observers knew the numerical agreement statistics at the time was not recorded, but these statistics were based on the independent readings and could not be influenced by the consensus. Multicenter work using shared definitions, a common diameter threshold, and comparable imaging protocols would do much to resolve the heterogeneity in this literature.

## 5. Conclusions

In this single-center sample of 603 adults, a canalis sinuosus that could be traced on CBCT was present in 53.2% of patients and was bilateral in 313 of these 321 patients (97.5%; 51.9% of the whole sample). At least one accessory canal was found in 31.7% of all patients and in 59.5% of those with a CS; 83.1% of the canals ended between the central incisor and canine regions, most often in the lateral incisor region (26.5%), and 63.4% ran straight vertically. When CBCT is available before implant placement, apical surgery, or other procedures in the anterior maxilla, the cross-sectional images should be examined for the CS and its accessory canals at the planned site, particularly at lateral incisor and canine sites, and the procedure should be planned to keep a margin from any canal identified. Because canals narrower than about 0.5 mm are not reliably detected, a negative scan does not exclude neurovascular tissue. The associations with age and sex were exploratory and need confirmation, and the risk of complications was not assessed.

## Figures and Tables

**Table 1:** Demographic characteristics of the study population and prevalence of canalis sinuosus and accessory canals.

Variable	*n*/*N*	Value	95% CI
Patients included, *n*	—	603	—
Age (years), mean ± SD (median; IQR; range)	603	46.2 ± 14.7 (48.0; 36.5–57.0; 18–83)	—
Male, *n* (%)	317/603	52.6	48.6–56.5
Female, *n* (%)	286/603	47.4	43.5–51.4
CS present (either side), *n* (%)	321/603	53.2	49.2–57.2
CS bilateral, *n* (%) ^a^	313/321	97.5	95.2–98.7
CS bilateral, all patients, *n* (%)	313/603	51.9	47.9–55.9
CS present, right side, *n* (%)	316/603	52.4	48.4–56.4
CS present, left side, *n* (%)	318/603	52.7	48.7–56.7
CS on one side only, right/left, *n* ^b^	—	3/5	—
Right vs. left CS, *p* ^b^	—	0.727	—
AC present (either side), *n* (%)	191/603	31.7	28.1–35.5
AC present among CS-positive patients, *n* (%) ^c^	191/321	59.5	54.0–64.7
AC bilateral, *n* (%) ^d^	83/191	43.5	36.6–50.5
AC bilateral, all patients, *n* (%)	83/603	13.8	11.2–16.7
AC present, right side, *n* (%)	129/603	21.4	18.3–24.8
AC present, left side, *n* (%)	145/603	24.0	20.8–27.6
AC on one side only, right/left, *n* ^b^	—	46/62	—
Right vs. left AC, *p* ^b^	—	0.149	—
Total accessory canals identified, *n*	—	385	—
AC count per AC-positive patient, mean ± SD (median; range)	191	2.0 ± 1.2 (1; 1–6)	—

SD standard deviation; IQR interquartile range; CI confidence interval (Wilson score method); CS canalis sinuosus; AC accessory canal. ^a^ Among CS-positive patients. ^b^ Exact McNemar test, right vs. left; the test uses only the patients with a canal on one side only. ^c^ AC requires anatomical continuity with the CS; all AC-positive patients were CS-positive. ^d^ Among AC-positive patients. Figures are per patient (*n* = 603), except in the rows for the right or left side, which count sides (603 of each).

**Table 2:** Prevalence of canalis sinuosus and accessory canals by sex and age group.

Group	*n*	CS, *n* (%)	95% CI	AC, *n* (%)	95% CI
Sex					
Male	317	172 (54.3)	48.8–59.7	108 (34.1)	29.1–39.4
Female	286	149 (52.1)	46.3–57.8	83 (29.0)	24.1–34.5
Comparison, *p* ^a^; *q* ^b^	—	0.624; 0.624		0.190; 0.253	
Age group, years					
≤29	111	51 (45.9)	37.0–55.2	27 (24.3)	17.3–33.1
30–39	62	27 (43.5)	31.9–55.9	14 (22.6)	14.0–34.4
40–49	149	78 (52.3)	44.4–60.2	49 (32.9)	25.9–40.8
50–59	170	104 (61.2)	53.7–68.2	67 (39.4)	32.4–46.9
≥60	111	61 (55.0)	45.7–63.9	34 (30.6)	22.8–39.7
Comparison, *p* ^a^; *q* ^b^	—	0.056; 0.113		0.040; 0.113	
Linear trend, *p* ^c^ (departure, *p*)	—	0.017 (0.321)		0.034 (0.140)	

^a^ Fisher’s exact test (sex) or Pearson’s chi-square (age group), unadjusted. ^b^ Benjamini–Hochberg *q* across the 4 comparisons; none reached *q* < 0.05. ^c^ Cochran–Armitage test for linear trend across the five ordered age groups, added post hoc and unadjusted; the test for departure from linearity is given in parentheses. CI, confidence interval (Wilson score method); CS, canalis sinuosus; AC, accessory canal. All figures are per patient (*n* = 603); a patient with a canal on either side was counted once.

**Table 3:** Diameter of the canalis sinuosus and accessory canals.

Structure	Group	*n*	Mean ± SD, mm	Median (IQR), mm	Range, mm
CS diameter	Right side	316	1.30 ± 0.24	1.23 (1.12–1.44)	0.87–2.11
CS diameter	Left side	318	1.31 ± 0.26	1.23 (1.12–1.43)	0.83–2.21
CS diameter	Male ^a^	340	1.41 ± 0.26	1.36 (1.22–1.55)	0.83–2.21
CS diameter	Female ^a^	294	1.18 ± 0.17	1.16 (1.06–1.28)	0.85–1.89
Left − right CS difference ^b^	Paired sides	313	0.003 (95% CI −0.017 to 0.023)	—	—
AC diameter	All canals	385	0.81 ± 0.18	0.79 (0.68–0.89)	0.51–1.63
AC diameter	Right side	183	0.83 ± 0.18	0.80 (0.68–0.91)	0.53–1.38
AC diameter	Left side	202	0.80 ± 0.18	0.76 (0.69–0.87)	0.51–1.63

SD standard deviation; IQR interquartile range. CS diameters are per side (634 CS-positive sides in 321 patients) and AC diameters per canal (385 canals in 191 patients); sides and canals from the same patient are not independent, and the adjusted comparisons in Table 5 take this into account. ^a^ CS-positive sides, grouped by patient sex. ^b^ Among bilateral CS; paired *t p* = 0.754, Wilcoxon *p* = 0.495.

**Table 4:** Localization and orientation of accessory canals according to side.

Category	Right Side, *n* (%)	Left Side, *n* (%)	Total, *n* (%)
Terminal localization			
Central incisor region	45 (24.6)	43 (21.3)	88 (22.9)
Between central and lateral incisor	38 (20.8)	27 (13.4)	65 (16.9)
Lateral incisor region	48 (26.2)	54 (26.7)	102 (26.5)
Canine region	25 (13.7)	40 (19.8)	65 (16.9)
First premolar region	1 (0.5)	9 (4.5)	10 (2.6)
Lateral to incisive foramen	9 (4.9)	7 (3.5)	16 (4.2)
Posterior to incisive foramen	12 (6.6)	20 (9.9)	32 (8.3)
Other localization ^a^	5 (2.7)	2 (1.0)	7 (1.8)
Total	183 (100)	202 (100)	385 (100)
Orientation			
Straight vertical	122 (66.7)	122 (60.4)	244 (63.4)
Curved medial	22 (12.0)	28 (13.9)	50 (13.0)
Curved lateral	24 (13.1)	19 (9.4)	43 (11.2)
Curved distal	15 (8.2)	33 (16.3)	48 (12.5)
Total	183 (100)	202 (100)	385 (100)

Percentages are within-side column percentages (*n* = 183 right, 202 left). The unit of analysis is the canal; the 385 canals came from 191 patients, and canals from the same patient are not independent, so the side distributions were not tested. ^a^ Terminations outside the seven regions of the de Oliveira-Santos et al. classification [35] were recorded as ‘other localization’ in this study.

**Table 5:** Clustered robust GEE analysis of canalis sinuosus and accessory canal presence and diameter.

Outcome (Model)	Term	Effect	95% CI	*p*	*q*	Obs. (Clusters)
CS presence (side level)	Age (linear + quadratic) ^a^	χ^2^(2) = 8.41	—	0.015	0.040	1206 (603)
	Male (ref: female)	OR = 1.02	0.73–1.42	0.912	0.987	
	Left side (ref: right)	OR = 1.01	0.98–1.05	0.480	0.641	
AC presence (side level)	Age (linear + quadratic) ^a^	χ^2^(2) = 8.98	—	0.011	0.040	1206 (603)
	Male (ref: female)	OR = 1.17	0.84–1.64	0.352	0.705	
	Left side (ref: right)	OR = 1.17	0.96–1.42	0.125	0.326	
CS diameter, mm (CS-positive sides)	Age (per 10 years)	*β* = 0.000	−0.014 to 0.014	0.959	0.987	634 (321)
	Male (ref: female)	*β* = 0.227	0.183–0.271	<0.001	<0.001	
	Left side (ref: right)	*β* = 0.004	−0.016 to 0.024	0.712	0.712	
AC diameter, mm (canal level)	Age (per 10 years)	*β* = 0.000	−0.018 to 0.019	0.987	0.987	385 (191)
	Male (ref: female)	*β* = 0.010	−0.043 to 0.063	0.713	0.987	
	Left side (ref: right)	*β* = −0.024	−0.058 to 0.010	0.163	0.326	

GEE fitted with independence working correlation and patient-clustered robust standard errors (accessory-canal diameter model clustered canals at patient level). OR odds ratio; *β* coefficient (mm); *q* Benjamini–Hochberg-adjusted *p*, computed separately for the eight age and sex terms and for the four side terms. Side terms are asymptotic Wald tests and therefore differ from the exact McNemar *p* values in Table 1. ^a^ Linear + quadratic age terms entered jointly; 2-df global Wald test (H0: both age coefficients = 0).

**Table 6:** CBCT studies that applied a minimum diameter of 0.5 mm: methods and patient-level prevalence.

Study, Country	Patients, *n*	Voxel, mm	Minimum Diameter	CS, %	AC, %
This study, Türkiye	603	0.125	CS and AC ≥ 0.5 mm; AC continuous with CS	53.2	31.7 (59.5) ^a^
Beckenstrater et al. [29], South Africa	500	≤0.4	CS and AC ≥ 0.5 mm; AC continuous with CS	99.6	58.8
Tetik and Zafersoy Akarslan [21], Türkiye	568	0.4	AC ≥ 0.5 mm	100	41.9
Tercanli Alkis et al. [13], Türkiye	248	0.32	AC ≥ 0.5 mm	100	35.5
Ghandourah et al. [19], Germany	201 ^b^	0.08–0.25	Canals ≥ 0.5 mm	100	67.6
Wu et al. [20], Taiwan	240	0.2	AC ≥ 0.5 mm and ≥1.0 mm ^c^	NR	80.4 and 51.7

CS, canalis sinuosus; AC, accessory canal; NR, not reported. Voxel sizes and thresholds are those stated in each study; prevalence is per patient and refers to the whole sample unless indicated. ^a^ Among CS-positive patients. ^b^ Adults; an adolescent subgroup was analyzed separately. ^c^ Diameter measured at the canal opening.

**Table 7:** Findings of this study and the clinical inferences drawn from them.

Observed in This Study	Clinical Inference (Not Tested in This Study)
CS traceable on CBCT in 53.2% of patients; bilateral in 97.5% of these (Table 1)	When a CS is identified on one side, the other side should be examined as well; failure to trace a CS does not exclude the anterior superior alveolar neurovascular bundle.
At least one AC in 31.7% of all patients and in 59.5% of CS-positive patients; 2.0 canals per AC-positive patient on average (Table 1)	Once a CS is seen, its branches should be sought specifically, and more than one may be present.
167 of 385 ACs (43.4%) ended in the lateral incisor or canine region; 19.9% of patients had at least one such canal (Table 4; Section 3.4)	Before implant placement or apical surgery at lateral incisor and canine sites, the palatal and interradicular bone should be checked on cross-sectional images for a canal in the planned path.
141 of 385 ACs (36.6%) curved medially, laterally, or distally (Table 4)	A canal may cross the planned implant axis obliquely and should be followed through consecutive sections rather than judged on a single slice.
AC diameters 0.51–1.63 mm; canals below 0.5 mm not detected (Table 3; Supplementary Table S4)	A negative CBCT does not exclude a canal; unexplained postoperative neurosensory symptoms warrant re-evaluation [52].
No distances to planned implant sites and no surgical outcomes were recorded	No safety distance has been validated; a margin of at least 2 mm is a pragmatic extrapolation [23,53,54], and guided or navigated placement, a change in implant position or length, or a non-implant restoration may be considered when it cannot be kept [55,56].

CS, canalis sinuosus; AC, accessory canal. The left column summarizes results of this study; the right column gives inferences that were not tested here and are offered as guidance for planning.

## Data Availability

Data available on request due to restrictions (e.g., privacy, legal or ethical reasons).

## Supplementary Materials

The following supporting information can be downloaded at https://www.mdpi.com/article/10.3390/diagnostics16213437/s1, Table S1: STROBE Statement—Checklist for Cross-Sectional Studies; Table S2: Patient-level logistic regression and random-intercept models of canal presence; Table S3: Agreement between the two perpendicular accessory canal diameters; Table S4: Accessory canal prevalence under stricter diameter thresholds.

## Abbreviations

The following abbreviations are used in this manuscript:

AC	Accessory canal
CBCT	Cone-beam computed tomography
CI	Confidence interval
CS	Canalis sinuosus
FOV	Field of view
GEE	Generalized estimating equations
ICC	Intraclass correlation coefficient
IQR	Interquartile range
OR	Odds ratio
STROBE	Strengthening the Reporting of Observational Studies in Epidemiology
