# Supplementary material for: Prevalence and Morphometric Features of the Canalis Sinuosus and Its Accessory Canals in Turkish Adult Patients: A Single-Center Cross-Sectional CBCT Study
Source: Diagnostics (Basel). 2026 Oct 22;16(21):3437. doi: 10.3390/diagnostics16213437 (PMC13761830; doi:10.3390/diagnostics16213437)
Supplement: Supplementary file 1 [file diagnostics-16-03437-s001.zip › diagnostics-4619634-supplementary.pdf]

Table S1. STROBE Statement—Checklist for Cross-Sectional Studies

Each item is located by the numbered sections, tables, and figures of the manuscript rather than by page, so the references hold after final typesetting.

Item	Recommendation	Location in the manuscript
**Title and abstract**		
**1(a)**	Indicate the study's design with a commonly used term in the title or the abstract.	Title; Abstract
**1(b)**	Provide in the abstract an informative and balanced summary of what was done and what was found.	Abstract, structured as Background/Objectives, Methods, Results and Conclusions
**Introduction**		
**2**	Explain the scientific background and rationale for the investigation being reported.	Section 1
**3**	State specific objectives, including any prespecified hypotheses.	Section 1, final paragraph
**Methods**		
**4**	Present key elements of study design early in the paper.	Title and Abstract; Section 2.1, opening sentence, which states the retrospective cross-sectional design, and the following paragraphs, which set out the setting, eligibility assessment, and adherence to STROBE
**5**	Describe the setting, locations, and relevant dates, including periods of recruitment, exposure, follow-up, and data collection.	Section 2.1 for the single center and the 2021–2024 record period; Section 2.2 for image acquisition
**6(a)**	Give the eligibility criteria, and the sources and methods of selection of participants.	Section 2.1, including the listed inclusion and exclusion criteria, and Figure 1; the reason for the age limit and the definitions of artifacts and incomplete imaging are also given in Section 2.1
**7**	Clearly define all outcomes, exposures, predictors, potential confounders, and effect modifiers. Give diagnostic criteria, if applicable.	Sections 2.3 and 2.4, where each outcome is defined together with its diagnostic criteria; the predictors are specified in Section 2.5
**8***	For each variable of interest, give sources of data and details of methods of assessment (measurement). Describe comparability of assessment methods if there is more than one group.	Sections 2.2 to 2.4. Both observers worked from the same acquisition parameters, viewing conditions, and measurement protocol, so the assessments are directly comparable
**9**	Describe any efforts to address potential sources of bias.	Section 2.1 for consecutive screening and the hierarchical exclusion rule; Section 2.3 for anonymization, observer blinding, independent duplicate reading, and reliability testing; Sections 4.2 and 4.3 for possible selection, measurement, and consensus-related bias

Item	Recommendation	Location in the manuscript
**10**	Explain how the study size was arrived at.	Section 2.1, minimum sample size calculation
**11**	Explain how quantitative variables were handled in the analyses. If applicable, describe which groupings were chosen and why.	Section 2.5. Age was modeled continuously with linear and quadratic terms; the five age categories were used for the descriptive tables, the subgroup comparisons, and the Cochran–Armitage trend test with equally spaced scores
**12(a)**	Describe all statistical methods, including those used to control for confounding.	Section 2.5, including the reason for using population-averaged (GEE) models and the units of analysis
**12(b)**	Describe any methods used to examine subgroups and interactions.	Section 2.5, covering the subgroup comparisons by sex and age group and the false discovery rate control applied within three prespecified families; the analyses added during peer review are listed separately in the same section
**12(c)**	Explain how missing data were addressed.	Section 2.1 and Figure 1: records that did not meet the eligibility criteria were removed at screening, and Table 1 reports age and sex for all 603 patients analyzed
**12(d)**	If applicable, describe analytical methods taking account of sampling strategy.	Section 2.1 for consecutive screening of the available records; Section 2.5 for the GEE approach adopted because both sides belong to the same patient
**12(e)**	Describe any sensitivity analyses.	Section 2.5, Wilcoxon signed-rank test, patient-level logistic regression, random-intercept models, and recalculation of accessory canal prevalence at stricter diameter thresholds (Supplementary Tables S2 and S4)
**Results**		
**13(a)**	Report numbers of individuals at each stage of study—e.g., numbers potentially eligible, examined for eligibility, confirmed eligible, included in the study, and analysed.	Section 2.1; Figure 1
**13(b)**	Give reasons for non-participation at each stage.	Section 2.1 and Figure 1, where every exclusion reason is listed with its number, in the order in which the reasons were applied; only the first reason that applied was recorded for each record
**13(c)**	Consider use of a flow diagram.	Figure 1
**14(a)**	Give characteristics of study participants and information on exposures and potential confounders.	Section 3, opening paragraph; Table 1

Item	Recommendation	Location in the manuscript
**14(b)**	Indicate the number of participants with missing data for each variable of interest.	Table 1, which reports the denominator for each variable; age and sex were available for all 603 patients
**15***	Report numbers of outcome events or summary measures.	Sections 3.2 to 3.4; Tables 1 to 4
**16(a)**	Give unadjusted estimates and, if applicable, confounder-adjusted estimates and their precision (e.g., 95% confidence interval). Make clear which confounders were adjusted for and why.	Sections 3.2 to 3.5 and Tables 1 to 5, giving crude figures with 95% confidence intervals alongside GEE estimates adjusted for age, sex, and side; patient-level odds ratios are given in Supplementary Table S2
**16(b)**	Report category boundaries when continuous variables were categorized.	Section 2.5 and Table 2; the age categories are ≤29, 30–39, 40–49, 50–59, and ≥60 years
**16(c)**	If relevant, consider translating estimates of relative risk into absolute risk for a meaningful time period.	Not applicable; the study reports prevalence and morphometric measures rather than risk estimates over a defined time period
**17**	Report other analyses done—e.g., analyses of subgroups and interactions, and sensitivity analyses.	Section 3.2 for the trend test across age groups; Section 3.3 for the Wilcoxon signed-rank test, the agreement between the perpendicular accessory canal diameters (Supplementary Table S3), and the stricter thresholds (Supplementary Table S4); Section 3.5 for the patient-level logistic regression, the random-intercept models, and the model restricted to CS-positive patients (Supplementary Table S2)
**Discussion**		
**18**	Summarise key results with reference to study objectives.	Section 4, opening paragraph
**19**	Discuss limitations of the study, taking into account sources of potential bias or imprecision. Discuss both direction and magnitude of any potential bias.	Section 4.3
**20**	Give a cautious overall interpretation of results considering objectives, limitations, multiplicity of analyses, results from similar studies, and other relevant evidence.	Sections 4 and 5
**21**	Discuss the generalisability (external validity) of the study results.	Section 4.2
**Other information**		
**22**	Give the source of funding and the role of the funders for the present study and, if applicable, for the original study on which the present article is based.	Funding statement in the back matter: the research received no external funding

** Give information separately for relevant groups where applicable.*

Source: STROBE Statement (www.strobe-statement.org).

Table S2. Patient-level logistic regression and random-intercept models of canal presence

Term	OR	95% CI	*p*
**A. Any CS, patient level (n = 603; 321 with a CS)**			
Age, linear term (per 10 years)[a]	1.10	0.98–1.24	0.100
Age, quadratic term[a]	0.94	0.88–1.01	0.075
Age, joint test	$\chi^2(2) = 7.66$	—	0.022
Male (ref: female)	1.01	0.73–1.41	0.935
**B. Any AC, patient level (n = 603; 191 with an AC)**			
Age, linear term (per 10 years)[a]	1.09	0.96–1.24	0.194
Age, quadratic term[a]	0.92	0.85–0.99	0.029
Age, joint test	$\chi^2(2) = 7.32$	—	0.026
Male (ref: female)	1.19	0.83–1.69	0.342
**C. Any AC among CS-positive patients, patient level, post hoc (n = 321; 191 with an AC)**			
Age, linear term (per 10 years)[a]	1.02	0.86–1.21	0.791
Age, quadratic term[a]	0.93	0.84–1.03	0.164
Age, joint test	$\chi^2(2) = 2.27$	—	0.321
Male (ref: female)	1.32	0.83–2.09	0.244
**D. AC presence, side level, random-intercept model, post hoc (1206 sides in 603 patients)[b]**			
Age, linear term (per 10 years)[a]	1.27	1.00–1.61	0.053
Age, quadratic term[a]	0.87	0.76–1.00	0.052
Age, joint test	$\chi^2(2) = 8.94$	—	0.011
Male (ref: female)	1.36	0.70–2.63	0.360
Left side (ref: right)	1.35	0.92–1.97	0.125
Between-patient SD of the random intercept (logit scale)	2.82	—	—

OR, odds ratio; CI, confidence interval; CS, canalis sinuosus; AC, accessory canal; SD, standard deviation. [a]Age was centered at the sample mean (46.2 years) and scaled per 10 years; the OR of the linear term is the odds ratio for a 10-year difference at the mean age, and an OR below 1 for the quadratic term indicates a curve that flattens and turns downward at older ages. Models A–C have one observation per patient and use robust (sandwich) standard errors. [b]Random-intercept logistic model fitted by maximum likelihood with Gauss–Hermite quadrature; its odds ratios are conditional on the patient and are therefore larger in magnitude than the population-averaged GEE estimates in Table 5 of the manuscript. For CS presence, the corresponding model was degenerate: because the two sides were concordant in 595 of the 603 patients, the between-patient SD was estimated at about 60 on the logit scale, and the subject-specific estimates were uninformative. Models C and D were added post hoc during peer review.

Table S3. Agreement between the two perpendicular accessory canal diameters

Measure (385 canals in 191 patients)	Value
First diameter (X), mean ± SD; median (IQR); range, mm	0.81 ± 0.19; 0.77 (0.69–0.89); 0.47–1.56
Second diameter (Y), mean ± SD; median (IQR); range, mm	0.82 ± 0.19; 0.79 (0.68–0.92); 0.48–1.69
Mean difference X − Y, mm (95% CI)[a]	−0.007 (−0.018 to 0.005); $p = 0.251$
SD of the differences, mm	0.123
95% limits of agreement, mm	−0.25 to 0.23
Absolute difference, median (IQR), mm	0.08 (0.05–0.13)
Absolute difference ≤ 0.125 mm (one voxel), n (%)	278 (72.2)
Absolute difference ≤ 0.25 mm (two voxels), n (%)	374 (97.1)
Ratio of the larger to the smaller diameter, median (IQR)	1.11 (1.07–1.18)
Ratio ≥ 1.20, n (%)	75 (19.5)
ICC, two-way, absolute agreement, single measures (95% CI)[b]	0.79 (0.75–0.83)

SD, standard deviation; IQR, interquartile range; CI, confidence interval; ICC, intraclass correlation coefficient. X and Y are the two perpendicular diameters measured on the same axial section; their mean was used as the canal diameter. [a]Confidence interval from an intercept-only GEE model with the patient as the cluster. [b]Percentile confidence interval from 2000 bootstrap resamples of patients. Six canals had one diameter below 0.5 mm (minimum 0.47 mm), but all 385 had a mean diameter of at least 0.5 mm. Post hoc analysis added during peer review.

Table S4. Accessory canal prevalence under stricter diameter thresholds

Minimum mean diameter	Canals retained, n (% of 385)	Patients with ≥ 1 AC, n (%; 95% CI); n = 603	CS-positive patients with ≥ 1 AC, n (%; 95% CI); n = 321
0.5 mm (study threshold)	385 (100)	191 (31.7; 28.1–35.5)	191 (59.5; 54.0–64.7)
0.6 mm	366 (95.1)	182 (30.2; 26.7–34.0)	182 (56.7; 51.2–62.0)
0.7 mm	264 (68.6)	146 (24.2; 21.0–27.8)	146 (45.5; 40.1–51.0)
0.8 mm	182 (47.3)	106 (17.6; 14.7–20.8)	106 (33.0; 28.1–38.3)
1.0 mm	55 (14.3)	35 (5.8; 4.2–8.0)	35 (10.9; 7.9–14.8)

AC, accessory canal; CS, canalis sinuosus; CI, confidence interval (Wilson method). Post hoc analysis added during peer review. Thresholds were applied to the mean of the two perpendicular diameters of each canal; canals below 0.5 mm were not recorded, so lower thresholds cannot be evaluated. When the larger of the two diameters was used instead, 43 patients (7.1%; 95% CI 5.3–9.5) had at least one canal of ≥ 1.0 mm. CS diameters ranged from 0.83 to 2.21 mm; a 1.0 mm threshold for the CS would have reduced its prevalence from 53.2% to 50.9% (307/603).
